# Giant nonlinear optical activity in two-dimensional palladium diselenide

**DOI:** 10.1038/s41467-021-21267-4

**Published:** 2021-02-17

**Authors:** Juan Yu, Xiaofei Kuang, Junzi Li, Jiahong Zhong, Cheng Zeng, Lingkai Cao, Zongwen Liu, Zhouxiaosong Zeng, Ziyu Luo, Tingchao He, Anlian Pan, Yanping Liu

**Affiliations:** 1 School of Physics and Electronics, Hunan Key Laboratory for Super-microstructure and Ultrafast Process, Central South University, Changsha, Hunan People’s Republic of China; 2 State Key Laboratory of High-Performance Complex Manufacturing, Central South University, Changsha, Hunan People’s Republic of China; 3 School of Electronics and Information, Hangzhou Dianzi University, Zhejiang, People’s Republic of China; 4 Key Laboratory of Optoelectronic Devices and Systems of Ministry of Education and Guangdong Province, College of Physics and Optoelectronic Engineering, Shenzhen University, Shenzhen, 518060 People’s Republic of China; 5 School of Chemical and Biomolecular Engineering, The University of Sydney, Sydney, NSW, 2006 Australia; 6 College of Materials Science and Engineering, Hunan University, Changsha, Hunan People’s Republic of China; 7 Shenzhen Research Institute of Central South University, Shenzhen, People’s Republic of China

**Keywords:** Materials for optics, Nanoscale materials

## Abstract

Nonlinear optical effects in layered two-dimensional transition metal chalcogenides have been extensively explored recently because of the promising prospect of the nonlinear optical effects for various optoelectronic applications. However, these materials possess sizable bandgaps ranging from visible to ultraviolet region, so the investigation of narrow-bandgap materials remains deficient. Here, we report our comprehensive study on the nonlinear optical processes in palladium diselenide (PdSe_2_) that has a near-infrared bandgap. Interestingly, this material exhibits a unique thickness-dependent second harmonic generation feature, which is in contrast to other transition metal chalcogenides. Furthermore, the two-photon absorption coefficients of 1–3 layer PdSe_2_ (*β* ~ 4.16 × 10^5^, 2.58 × 10^5^, and 1.51 × 10^5^ cm GW^−1^) are larger by two and three orders of magnitude than that of the conventional two-dimensional materials, and giant modulation depths (*α*_s_ ~ 32%, 27%, and 24%) were obtained in 1–3 layer PdSe_2_. Such unique nonlinear optical characteristics make PdSe_2_ a potential candidate for technological innovations in nonlinear optoelectronic devices.

## Abstract (web summary)

Strong nonlinearities in 2D materials can lead to interesting applications in optoelectronics. Here the authors investigate the optical nonlinearity of palladium diselenide, determine the layer dependent two photon absorption efficiency and the saturable absorption modulation depth.

## Introduction

Nonlinear optical (NLO) properties play an increasingly significant role in the development of laser technology, optical spectroscopy, and material structure analysis methodologies^1–4^. Recently, the study of NLO activity in two-dimensional (2D) layered materials (such as graphene, transition metal chalcogenides (TMDCs), and black phosphorus (BP)) has made significant progress^5–12^. The extraordinary nonlinearities of graphene, including saturable absorption (SA)^13,14^, optical limiting^15^ and harmonic generation^16^, have attracted great interest. However, the inherent properties of graphene, like its zero-bandgap and centrosymmetric crystal structure^17^, hinder the study of its NLO effects, including the second harmonic generation (SHG) and multiphoton absorption features. On the contrary, BP possesses intrinsic anisotropy, strong light-matter interaction, layer-dependent direct bandgap (0.3–2 eV), and a wide range of adjustable optical response^18,19^, leading to high SA modulation depths (~27.6% at 400 nm, ~12.4% at 800 nm^20^) and a large two photon absorption (TPA) coefficient ((−6.98 ± 0.6) × 10^3^ cm GW^−1^ ^21^). However, BP lacks sufficient air stability, resulting in the rapid degradation of its NLO properties. Meanwhile, the most studied TMDC materials (MX_2_, M = Mo, W, and X = S, Se, Te) that exhibit various fascinating NLO activities, such as SHG, SA and TPA, have sizable bandgaps ranging from visible to the ultraviolet region (1–2.5 eV) and there is a shortage of narrow-bandgap materials^22^. Interestingly, PdSe_2_ is a pentagonal 2D material with robust anisotropy, high carrier mobility, and air stability, that give it considerable advantages in nonlinear optoelectronic devices^23^. In addition, PdSe_2_ has a modifiable thickness-dependent bandgap from 0.03 to 1.37 eV^24^, filling the interspace between zero-gap graphene and large-gap TMDCs. Meanwhile, Xu et al. demonstrated the ultrafast SA performance of liquid-phase exfoliated PdSe_2_ that generated the dissipative soliton and pulse energy^25^. Zhang et al. experimentally investigated the mode-locked Er and Yb-doped operations in multilayer PdSe_2_^26^. These works demonstrate the capacity of PdSe_2_ for broadband ultrafast photonic devices. However, to date, the current experimental investigation of the NLO activities of PdSe_2_ (especially the monolayer sample) is insufficient. Therefore, an extensive and in-depth study of the NLO behavior of PdSe_2_ is of great significance for the expansion of its optoelectronic applications.

Herein, we report the fabrication of large-size PdSe_2_ flakes with different layers and present a comprehensive investigation of thickness-dependent NLO properties of the PdSe_2_ flakes. Extraordinarily, owing to its unique layer-dependent inversion symmetry, PdSe_2_ exhibits a SHG behavior that is totally opposed to that in other 2D materials^27,28^. Moreover, the PdSe_2_ flakes exhibit giant TPA coefficients (*β*) that are two to three orders of magnitude larger compared to that of the conventional semiconductors^29–31^. Importantly, excellent saturable absorption was observed in 1–3 layer (L) PdSe_2_, with much higher modulation depths (*α*_s_) than that of the most other 2D materials^32–34^. These findings not only provide profound insights into the NLO features of this innovative nanomaterial, but also open an avenue for future nonlinear optoelectronic applications.

## Results

### Crystal structure and characterization of few-layer PdSe_2_ flakes

**Fig. 1:**
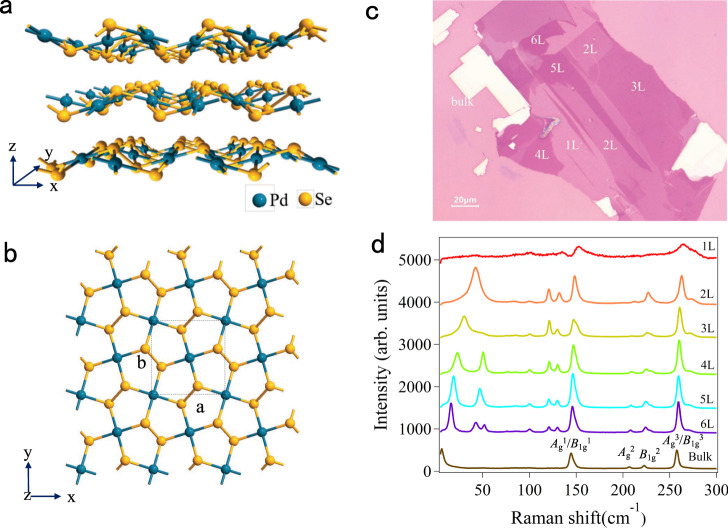
Crystallographic structure and optical characteristics of a few-layer PdSe_2_. **a** The lattice structure of PdSe_2_ flakes with a puckered pentagonal. The unit cell vectors are denoted by the x, y, and z. Blue and yellow globes denote the Pd and Se atoms, respectively. **b** Top view of the atomic crystal structure of monolayer PdSe_2_. The black dotted frame represents a unit cell of PdSe_2_. The *a* and *b* denote the lattice constants along the x and y directions, respectively. **c** The optical microscope image of a few-layer PdSe_2_ on the silicon substrate with a 285 nm SiO_2_ layer. The numbers of sample layers containing 1–6 L were marked with white letters, and the scale bar is 20  μm. **d** Raman spectra of the 1–6 L and bulk PdSe_2_ flakes with the same intensity axis. The low-frequency Raman peaks (0−100 cm^−1^) were robust and even exceeded that of the high-frequency modes. The Raman vibration modes of bulk PdSe_2_ were marked as *A*_g_^1^, *A*_g_^2^, *A*_g_^3^, *B*_1g_^1^, *B*_1g_^2^, and *B*_1g_^3^ mode.

The unique optical characteristics of PdSe_2_ are correlated to its special structure. It has a stratiform structure that is identical to some of the other 2D materials (graphene, hexagonal boron nitride (hBN), BP, and TMDCs), as shown in Fig. 1a. The Pd and Se atoms combine to form a covalent bond within a monolayer, and the van der Waals (vdWs) force dominates the interactions between adjacent layers. In the side view, PdSe_2_ exhibits a unique plicate crystal structure with a monolayer vertical plicate height of 1.604 Å. In comparison to the traditional 2D materials with the highly symmetric hexagonal honeycomb structure, the Pd and Se atoms in PdSe_2_ constitute an asymmetric pentagonal structure (Fig. 1b). The ultrathin PdSe_2_ crystal displays robust interlayer coupling with a relatively large binding energy of 190 meV/atom^35^, as manifested in the process of mechanical exfoliation. Monolayer and few-layer PdSe_2_ flakes are difficult to be obtained by the conventional scotch tape method^23,36^. Instead, the gold-assisted exfoliation method was adopted to acquire the large-size monolayer and few-layer PdSe_2,_ as shown in Fig. 1c on the Si/SiO_2_ substrates. The height profiles of the PdSe_2_ samples were obtained by atomic force microscopy (AFM) measurements (Fig. S1 in Supplementary Information). According to the measurements, the average thickness of a single layer PdSe_2_ is 0.7 nm. Recently, Raman technology has been demonstrated as an excellent approach in determining the layer number of various 2D materials^37–40^. Through analyzing the Raman spectrum, the layer number of a thin sample can be identified quickly, precisely and non-destructively. The Raman spectra of the 1–6 L and bulk PdSe_2_ samples at a wavenumber range of 0–300 cm^−1^ were obtained to estimate the layer numbers (Fig. 1d). Obviously, the Raman peak position varied gradually as the layer number increased. It is worth mentioning that the Raman peaks in the low-frequency region (0–100 cm^−1^) varied by a large margin. The low-frequency Raman modes indicate the occurrence of interlayer vibrations in the samples that were robust, and even exceeded those of the high-frequency modes (100–300 cm^−1^), which were attributed to the strong interlayer coupling of PdSe_2_. The peak positions of the low-frequency Raman modes have a specific relationship with the layer numbers, as shown in Fig. S2 in Supplementary Information. Because of the significant shifts of the low-frequency Raman modes, the layer number could be determined precisely. The feasibility and specific operation of such approach have been reported in detail in our previous work^24^.

**Fig. 2:**
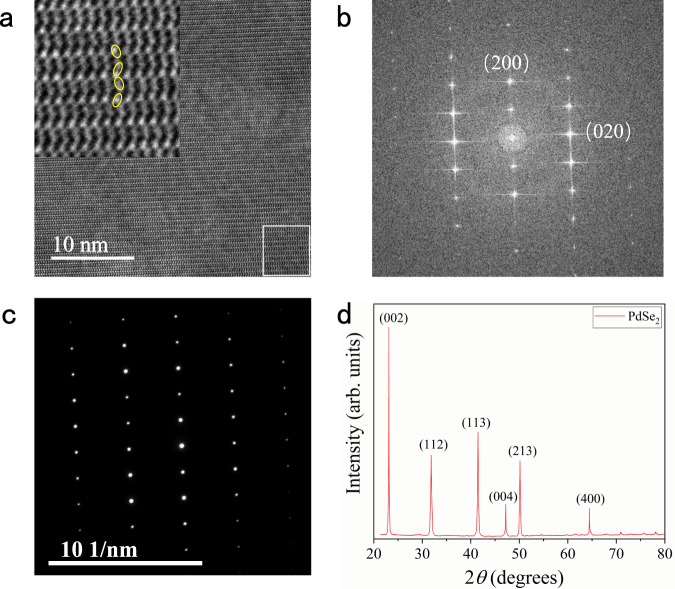
TEM and XRD characterizations of PdSe_2_ flakes. **a** HRTEM image of the few-layer region of PdSe_2_ flakes. The inset shows a zoom-in of the white square regions, revealing the atomic models. The scale is marked in the lower-left corner. **b** FFT diffraction patterns of the solid white areas highlighted on the image (**a**). **c** SAED pattern of the PdSe_2_. **d** XRD pattern of bulk PdSe_2_ is in good agreement with the standard PDF card (PDF#97-017-0327). The diffraction pattern exhibits six robust peaks at 23.1, 31.8, 41.5, 47.2, 50.14, and 64.4°, respectively.

Furthermore, the high-resolution transmission electron microscopy (HRTEM) characterization was performed to reveal the atomic structure of PdSe_2_. The periodic atomic arrangement of PdSe_2_ could be distinctly observed, and the sample had a flat surface (Fig. 2a). No obvious defects were observed, indicating high crystallinity of the samples. Figure 2b shows the fast Fourier transform (FFT) diffraction patterns. The distinct diffraction spots correspond to the two principle crystal planes of (200) and (020). The selected-area electron diffraction (SAED) pattern was taken from the few-layer region of PdSe_2_ (Fig. 2c), indicating the high-quality single-crystal structure and confirming the isometric system of PdSe_2_. For the further investigation of our experimentally prepared PdSe_2_, X-ray diffraction (XRD) characterization was performed. As shown in Fig. 2d, the diffraction pattern exhibits six robust peaks at 23.1, 31.8, 41.5, 47.2, 50.14, and 64.4°, corresponding to (002), (112), (113), (004), (213), and (400) atomic planes of PdSe_2_, demonstrateing the high crystal quality of the sample^41^.

### SHG properties of PdSe_2_ flakes

**Fig. 3:**
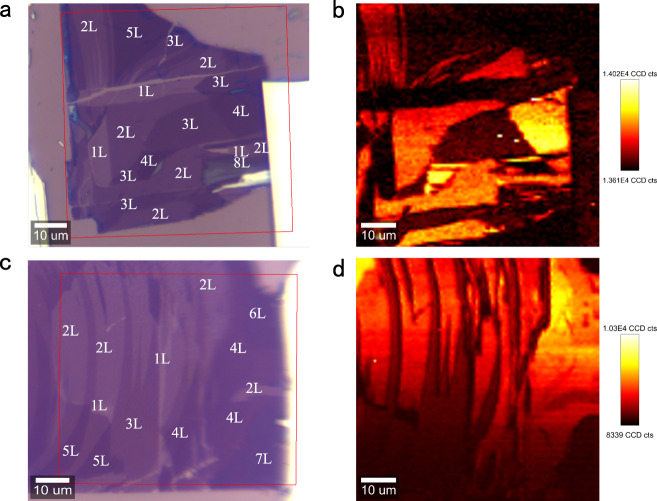
SHG spatial mapping of few-layer PdSe_2_. **a**, **c** The optical images of the PdSe_2_ flakes for the SHG characterization. The red boxes indicate the selected area for the SHG measurements. The layer numbers are labeled by the white numbers. The region elected in **a** contain 1–5 L and 8 L PdSe_2,_ and the area selected in **c** includes 1–7 L PdSe_2_, ensuring sufficient sampling. The PdSe_2_ samples were excited by 800 nm wavelength using 50× objective with a NA value of 0.55. **b**, **d** The spatial mapping of the integrated SHG signals corresponding to the regions in **a** and **c**, respectively. The intensities of the SHG signals are different for different layers. The SHG signal is almost absent in the odd-layer PdSe_2_, while it is strong in the even-layer PdSe_2_. The color bars are shown on the right of the figure.

To better understand the NLO characteristics of the PdSe_2_ flakes, the SHG properties of few-layer PdSe_2_ at the excitation wavelength of 800 nm were studied in detail. For the monolayer region, the detected SHG signal was negligible. When the laser spot was switched to the bilayer area, strong signals could be observed at 400 nm. In order to further demonstrate the SHG signal dependence on the PdSe_2_ layer number, the spatial mapping was implemented. The red box in Fig. 3a indicates the scanning regions of the sample, containing 1–5 L, 8 L, and bulk PdSe_2_ flakes. Clearly, the SHG signals from even-numbered layers of PdSe_2_ were strong, while the signals from odd-numbered layers and the bulk PdSe_2_ were almost negligible (Fig. 3b). In order to ensure sufficient sampling, spatial scanning of another sample (consisting of 1–7 L and bulk PdSe_2_) was carried out and the same results were obtained, as can be seen in Fig. 3c, d. Such characteristics are exactly opposite to the SHG phenomenon displayed by other group VI 2D materials, where a strong SHG signal was only observed from the odd-numbered layers, and no signal was detected from even-numbered layers^27,28,42^. It is well known that strong SHG signals will occur only in 2D materials with a broken inversion symmetry^43^. Interestingly, the odd-numbered layers of PdSe_2_ belong to the *C*_2h_ *(2/m)* point group and *P2*_1_*/c* (No.14) space group, which have an inversion symmetry, while the even-numbered layers possess a *C*_2v_ *(mm2)* point group and *Pca2*_1_ (No. 29) space group with a broken inversion symmetry^23^. Meanwhile, the bulk PdSe_2_ has an orthogonal structure with a *D*_2h_ point group and *Pbca* (No. 61) space group with an inversion symmetry^23^. These characteristics, that lead to the unique layer dependence of the SHG signal of PdSe_2_ flakes, are very unlike that of the other 2D materials that have an inversion symmetry in the even layers and a broken inversion symmetry in the odd layers^42,44^. Additionally, it was found that the PdSe_2_ samples were very uniform in thickness, attributing to the gold-assisted exfoliation method.

**Fig. 4:**
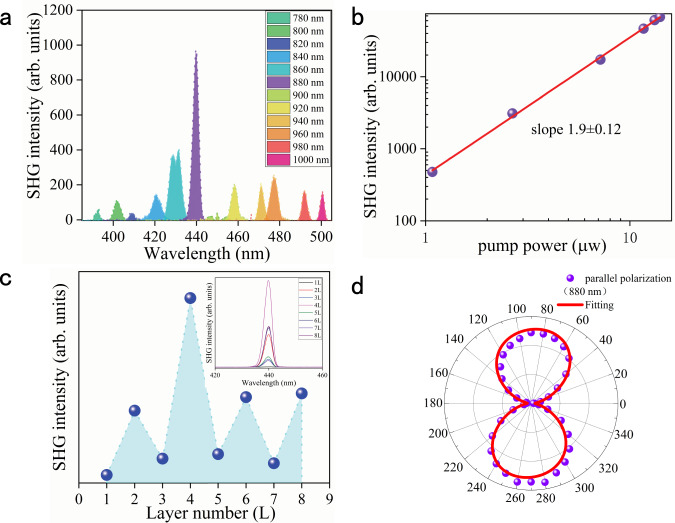
SHG characterization of the PdSe_2_ flakes. **a** The SHG intensities excited at various wavelengths from 780 to 1000 nm, represented by different colors. The highest intensity appeared at the wavelength of 440 nm, manifesting the strongest SHG signal of excitation at 880 nm. **b** The excitation power dependence of SHG intensities in 4 L PdSe_2_. Both axes are shown on the log scale. The correlation of the pump power and SHG intensities exhibits was linear with a slope of 1.9 ± 0.12. **c** The SHG signal excited at the fixed wavelength of 880 nm in 1–8 L PdSe_2_. The integrated intensity of SHG varied with different layers, represented by the blue dots. The SHG signal was the strongest in the 4 L sample. The inset represents the SHG intensities versus the layer numbers of PdSe_2_. **d** The purple dots indicate the polar plot of integrated SHG intensities excited by the 880 nm laser in 4 L PdSe_2_. The corresponding fitting result is denoted by the solid red curve.

Generally, there are many factors that can influence the SHG intensity of PdSe_2_, and among those the excitation wavelength is relatively a significant one. To determine the wavelength that could generate the highest SHG signal, the SHG spectra were acquired by changing the excitation wavelengths from 780 to 1000 nm at a step length of 20 nm. As shown in Fig. 4a, there were strong excitation wavelength-dependent optical signals. The highest optical signal was observed under 880 nm excitation, which could be caused by the SHG effect. To explore the physical mechanism under excitation at 880 nm, the correlation between the detected optical intensity and the excitation intensity was measured. As presented in Fig. 4b, it was found that the optical intensity was quadratically dependent on the excitation power, and this was definitely the SHG signal. In addition, when excited with the same wavelength, the layer-dependent SHG signals of PdSe_2_ were measured. As expected, the odd-numbered layers (1, 3, 5, and 7 L) exhibited very low SHG intensities while the even-numbered layers (2, 4, 6, and 8 L) displayed much stronger SHG signals. The SHG intensities as a function of the layer numbers were plotted in Fig. 4c, and it can be seen that the highest SHG intensity occurred in 4 L PdSe_2_ (inset). The SHG intensity of PdSe_2_ is mainly affected by two factors, the asymmetry and absorption intensity^28,45^. The odd-numbered layers of PdSe_2_ (1, 3, 5, and 7 L) reserve the symmetry so that no distinct SHG signal is detected. However, as the layer number increased, the signal was weak and non-zero. This was due to the small but finite optical phase shift between the PdSe_2_ layers^28^. Additionally, in the even-numbered layers (2, 4, 6, and 8 L) of PdSe_2_, as the layer number increased, the asymmetry of PdSe_2_ increased, which caused a strong SHG signal in 4 L PdSe_2_^46^. However, when the layer number was more than 4 L, the optical absorption intensity in PdSe_2_ became much stronger, leading to a lower collection efficiency of SHG^47^. Therefore, influenced by these two factors, the 4 L PdSe_2_ exhibited the strongest SHG intensity. In general, the NLO process in a crystal can be expressed by the relationship of polarization vector and the electric field of the incident light in the form of^1,2^:

1 $${\mathbf{P}} = \varepsilon _0\left( {\chi {\mathbf{E}} + \chi ^{\left( 2 \right)}{\mathbf{E}}^2 + \chi ^{\left( 3 \right)}{\mathbf{E}}^3 + \cdots } \right)$$

where ${\mathbf{P}}$ is the polarization vector, and $\varepsilon _0$ is the permittivity in a vacuum, while $\chi$ is the electric susceptibility of the medium, and ${\mathbf{E}}$ is the electric field component of the incident light. For the SHG process, the dominated term is ${\mathbf{E}}^2$. The second-order electric susceptibility, $\chi ^{\left( 2 \right)}$, reflects the SHG efficiency, and for 4 L PdSe_2,_ it was determined to be 5.17 × 10^−11^ m V^−1^ at the excitation of 880 nm, according to Eqs. S1–3 in the Supplementary Information (Note S1). The $\chi ^{\left( 2 \right)}$ values for different 2D materials are presented in Table S1. It can be seen that the $\chi ^{\left( 2 \right)}$ value of PdSe_2_ is comparable to that of the other 2D materials.

Besides, the polarization-resolved SHG signals were also acquired, with the aim of identifying the crystal orientation of the sample. In our measurement, the polarization orientations of the incident and detected lights were parallel (for more specific information, see Experimental details). The experimental configuration and the measured parameters of the SHG measurement are illustrated in Fig. S3 in the Supplementary Information. Under a parallel configuration, the polar plot of the SHG signals represented distinct anisotropy, embodied in a dumbbell shape (Fig. 4d). The SHG intensity had its minimum at 0°, then it gradually increased as the polarization orientation rotated, reaching the maximum at 90°. The slight skewing of the polar plot might be resulted from the distorted crystal lattice. The nonlinear dielectric susceptibility tensor was employed to analyze the polarization-resolved SHG signals. The SHG polarization vector, ${\mathbf{P}}$, is related to the electrical field, ${\mathbf{E}}$, of the incident light, and their relationship is expressed as^46^

2 $${\mathbf{P}} = d{\mathbf{E}}$$

where *d* is the dielectric susceptibility tensor. Under the three-dimensional geometric space, both ${\mathbf{P}}$ and ${\mathbf{E}}$ have three components in the x, y, and z directions. Likewise, other variables also have a three-dimensional form. Therefore, the above formula can be further expressed as follows

3 $$\left( {\begin{array}{*{20}{c}} {P_x} \\ {P_y} \\ {P_z} \end{array}} \right) = \left( {\begin{array}{*{20}{c}} {\begin{array}{*{20}{c}} {d_{11}} & {d_{12}} & {d_{13}} \\ {d_{21}} & {d_{22}} & {d_{23}} \\ {d_{31}} & {d_{32}} & {d_{33}} \end{array}} & {\begin{array}{*{20}{c}} {d_{14}} & {d_{15}} & {d_{16}} \\ {d_{24}} & {d_{25}} & {d_{26}} \\ {d_{34}} & {d_{35}} & {d_{36}} \end{array}} \end{array}} \right)\left( {\begin{array}{*{20}{c}} {\begin{array}{*{20}{c}} {E_xE_x} \\ {E_yE_y} \\ {E_zE_z} \end{array}} \\ {\begin{array}{*{20}{c}} {2E_yE_z} \\ {2E_xE_z} \\ {2E_xE_y} \end{array}} \end{array}} \right)$$

where *d*_*ij*_ is the element of the dielectric susceptibility tensor. For the experimental backscattering setup, the electrical field intensity of the incident light in the z-direction was negligible. Thus, the electrical field intensities in the x and y directions can be expressed as

4 $$\begin{array}{l}E_x = E_0\cos \theta \\ E_y = E_0\sin \theta \end{array}$$

where $E_0$ is the module of the electrical field intensity of the incident light, and $\theta$ is the angle between the *x*-axis of the crystal lattice of PdSe_2_ and the polarization orientation of the incident light. Under the parallel backscattering configuration, the eventual SHG intensity is only correlated with $P_x$ and $P_y$, expressed as

5 $$I_\parallel \propto \left( {P_x\cos \theta + P_y\sin \theta } \right)^2$$

where $I_\parallel$ is the detected SHG intensity under the parallel configuration. Substitute Eq. (3) with Eq. (5), we obtained

6 $$I_\parallel \propto \left[ \begin{array}{l}d_{11}\cos \theta ^3 + \left( {d_{21} + 2d_{16}} \right)\cos \theta ^2\sin \theta \\ \quad \quad \quad \quad + \,\left( {d_{12} + 2d_{26}} \right)\cos \theta \sin \theta ^2 + d_{22}\sin \theta ^3\end{array} \right]^2$$

Using this formula, the experimental SHG intensities were fitted appropriately, as shown by the solid red curve in Fig. 4d. It can be seen that the experimental results are entirely consistent with the theoretical analysis. Meanwhile, the polarization-resolved SHG measurements are expected to accurately identify the crystal orientation of PdSe_2_ at the macro scale.

### TPA properties of PdSe_2_ flakes

**Fig. 5:**
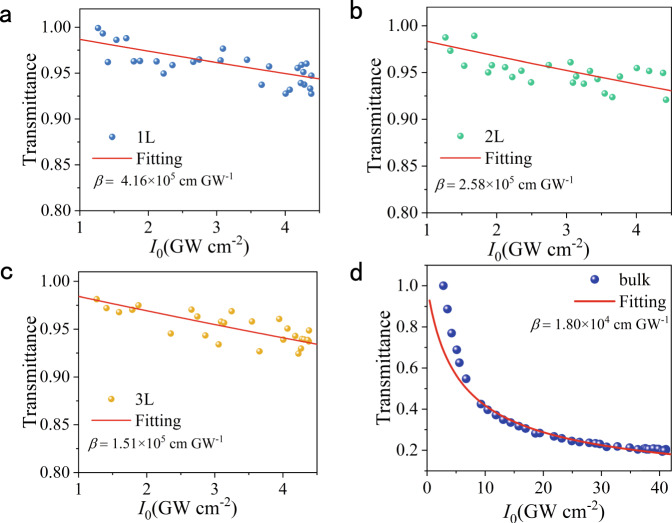
TPA properties of 1–3 L and bulk PdSe_2_ flakes. **a**–**c** Nonlinear transmittance of 1–3 L PdSe_2_. The blue, green, and yellow dots represent the experimental data, while the solid red lines show the fitted results using the TPA model. The TPA coefficients (*β*) of 1, 2, and 3 L PdSe_2_ are 4.16 × 10^5^, 2.58 × 10^5^, and 1.51 × 10^5^ cm GW^−1^, respectively. **d** Nonlinear transmittance of bulk PdSe_2_. The blue dots represent the experimental results, while the solid red lines show the fitted results using the TPA model. The TPA coefficient (*β*) of bulk PdSe_2_ is 1.80 × 10^4^ cm GW^−1^. All the above measurements were performed under 800 nm excitation.

The NLO processes in 2D materials can be divided into two classifications, the parametric and non-parametric process^48^. The parametric process in 2D materials is mainly a scattering process, where the ground state is excited into a virtual state, and there is no electron transfer and absorption between the two virtual states, such as the SHG above. On the contrary, the non-parametric process is the excitation from the ground state to a real state, which involves absorption and electron transfer, mainly including TPA and SA. To deeply explore the NLO properties of PdSe_2_, its TPA was further investigated. Figure S4 illustrates the experimental setting of the home-built nonlinear transmittance system used to perform the TPA and SA measurements, together with the measured parameters. To perform the TPA measurement, the monolayer and few-layer PdSe_2_ flakes, obtained from the bulk crystal by gold-assisted exfoliation, were transferred to the transparent quartz substrates. After that, their TPAs were measured using the nonlinear transmittance method. According to our previous reports, PdSe_2_ exhibits distinct linear dichroism features under the excitation of 300–800 nm^24^. When the excitation wavelength was 800 nm, the linear absorption coefficients of 1, 2, and 3 L PdSe_2_ were estimated to be 3.9 × 10^3^, 1.3 × 10^4^ and 1.9 × 10^4^ cm^−1^, respectively, according to the linear absorption measurement in Fig. S5 in Supplementary Information. Thus, the linear absorption was almost negligible in the solid film. Consequently, TPA could be predicted when excited by pulses at 800 nm. As intuitively described in Fig. 5, the transmittance of the PdSe_2_ flakes decreased with the increasing of the incident light intensity, exhibiting a typical TPA characteristic.

Based on the nonlinear transmittance method, the TPA coefficients of the samples could be determined by the following formula^49^:

7 $$T\left( {I_0} \right) = \frac{{I_{\mathrm{t}}}}{{I_0}} = \frac{{\left[ {\ln \left( {1 + I_0t\beta } \right)} \right]}}{{I_0t\beta }}$$

where $T(I_0)$ is the transmittance of the PdSe_2_ sample, and $I_{\mathrm{t}}$ is the transmitted light intensity of the sample, while $I_0$ is the incident irradiance intensity and *β* is the TPA coefficient with $t$ being the thickness of the PdSe_2_ sample. By fitting the experimental data with Eq. (7), the *β* values of 1, 2, 3 L, and bulk PdSe_2_ were determined to be 4.16 × 10^5^, 2.58 × 10^5^, 1.51 × 10^5^, and 1.80 × 10^4^ cm GW^−1^, respectively. These values are two to three orders of magnitude larger compared to those of other conventional semiconductors, such as MoS_2_, WS_2_, GaAs, CdS, and ZnO (Table S2 in Supplementary Information)^29–31,50^, meaning the great potential of the few-layer PdSe_2_ flakes for TPA-based applications. Such giant TPA coefficients might originate from the strong exciton effects in PdSe_2_^51^. This was enhanced by the strong dielectric screening owing to the ultrathin structure of the monolayer^23,52^. In addition, it was found that the TPA coefficient of PdSe_2_ gradually decreased with the increasing of layers. Such a phenomenon was mainly attributed to the fact that the bandgap decreased with the increase of the number of layers, and there was a certain detuning between the formative exciton and the two-photon energy^53^.

### SA properties of PdSe_2_ flakes

**Fig. 6:**
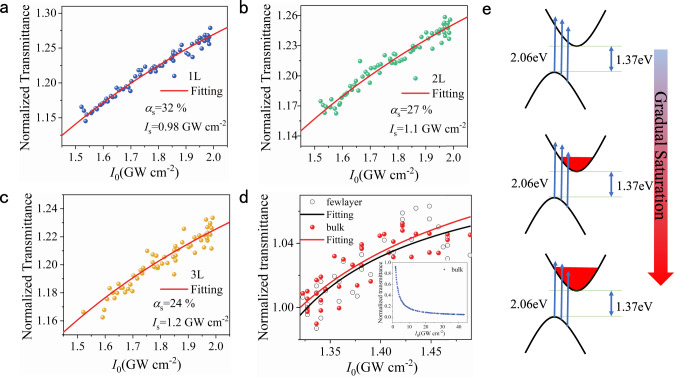
SA properties of 1–3 L and bulk PdSe_2_ flakes. **a**–**c** Nonlinear transmittance of 1–3 L PdSe_2_. The blue, green, and yellow dots represent the experimental data, while the solid red lines show the fitted results using the SA model. The SA intensities of 1, 2, and 3 L PdSe_2_ are 0.98, 1.1, and 1.2 GW cm^−2^, respectively. The modulation depths of 1, 2, and 3 L PdSe_2_ are 32%, 27%, and 24%, respectively. **d** Nonlinear transmittance of the bulk PdSe_2_. The white and red dots represent the experimental data, while the black and red solid lines show the fitting curves of few-layer and bulk PdSe_2_ using the SA model. The inset shows a reverse SA trend of the thick bulk PdSe_2_ with increased laser power. **e** The electronic transition process of SA. All the above measurements were performed under 600 nm excitation.

Considering that strong SA was widely reported in various two-dimensional TMDCs, including MoS_2_^54^, WS_2_^55^, WSe_2_^56^, and MoSe_2_^57^, a typical SA effect could also be expected in our PdSe_2_ in the case of 600 nm excitation. Figure 6 shows the trends of the normalized transmittance versus the incident intensity in 1–3 L and bulk PdSe_2_. The fitting could be performed according to a standard model of SA, as shown in the following formula^58^:

8 $$T\left( {I_0} \right) = 1 - \frac{{\alpha _{\mathrm{s}}}}{{1 + \frac{{I_0}}{{I_{\mathrm{s}}}}}} - \alpha _{\mathrm{u}}$$

where $\alpha _{\mathrm{s}}$ is the modulation depth, and $I_{\mathrm{s}}$ is the saturable intensity, while $\alpha _{\mathrm{u}}$ is the unsaturated absorption component. The obtained fitting parameters of the modulation depths (*α*_s_) of 1–3 L PdSe_2_ were 32%, 27% and 24%, respectively. The saturable intensities (usually the half of the highest optical absorption) of 1–3 L PdSe_2_ were 0.98, 1.1, and 1.2 GW cm^−2^, respectively. As the sample thickened, the saturable intensity gradually increased. The modulation depths are much higher than that of the most other 2D materials, such as SnSe^32^, MoSe_2_^33^, MoS_2_^34^, and PtSe_2_^59^, which are promising for the application in ultrafast lasers. The high modulation depths mainly resulted from the low unsaturated loss, indicating a low defect density in the PdSe_2_^7^. However, the modulation depths in (thinner) bulk and few-layer PdSe_2_ (more than 10 L, only 2.8%) were much lower than that in 1–3 L PdSe_2_, which might be attributed to the increased unsaturated loss induced by the strengthened scattering^7^. It is worth noting that there was no SA phenomenon for the thicker bulk PdSe_2_ since when the sample was thick enough, the light capacity was sufficient, and the saturation state would not be easily reached. The excellent SA property of 1–3 L PdSe_2_ endows its application prospect in the field of ultrafast lasers.

To understand the principle of the transmittance dependence on the incident irradiance, the electronic transition process in the momentum space should be clear. Figure 6e illustrates the electronic transition process of SA in PdSe_2_. The bandgap of the monolayer (bulk) PdSe_2_ was determined to be 1.37 (0.03) eV (Fig. S6 in Supplementary Information), indicating the unusual indirect bandgap characteristics. In our case, the energy of the incident photon (2.06 eV) was much higher than the bandgap of a few-layer PdSe_2_. For low excitation intensity, the electrons in the valence band jumped to the relatively high energy levels in the conduction band, as illustrated on the top panel of Fig. 6e. Then, the hot electrons relaxed to the lower energy band, and populated in the bottom of the conduction band. With the increase in incident optical intensity, the number of excited electrons also increased. Electron is a fermion, that cannot populate at the same energy level. Thus, the energy levels were gradually filled from the bottom to the top in the conduction band (middle panel in Fig. 6e). When the energy level of the conduction band was filled with electrons as the incident light intensity increased, the electronic transition process was impeded owing to the Pauli exclusion principle. The number of the absorbed photons then gradually approached saturation, as indicated on the bottom panel of Fig. 6e. This explains the gradually saturable transmittance when the incident optical intensity was progressively increased.

In summary, large-area PdSe_2_ flakes with different thicknesses were successfully fabricated on the Si/SiO_2_ and quartz substrates. Significantly, in contrast to that of the other 2D materials, robust broadband SHG properties in the even-numbered layers of PdSe_2_ were demonstrated, as a result of the unique anisotropic puckered pentagonal structure of PdSe_2_. Besides, the TPA and SA characteristics of the PdSe_2_ flakes were investigated. Under the excitation at 800 nm, 1–3 L and bulk PdSe_2_ exhibited giant TPA coefficients (*β*) of 4.16 × 10^5^, 2.58 × 10^5^, 1.51 × 10^5^, and 1.80 × 10^4^ cm GW^−1^, respectively, which are at least two orders of magnitude larger than those of the conventional semiconductors. Interestingly, when the excitation was switched to 600 nm, on account of the ultra-low unsaturable loss, strong SA with the modulation depths (*α*_s_) up to 32%, 27% and 24% for 1–3 L PdSe_2_ were observed. Compared to those of the other traditional 2D materials, these values are exceptionally large. Such prominent NLO activity of 2D PdSe_2_ provides great prospects for potential applications in optical switching, ultrafast lasers, saturation absorbers, optical limiters, and micro/nano optical modulation devices.

## Methods

### Sample preparation

The PdSe_2_ flakes were exfoliated from bulk single-crystal PdSe_2_ (HQ graphene) onto silicon and transparent quartz substrates, employing the gold-assisted exfoliation. The thicknesses of the 1, 2, 3 L, and bulk PdSe_2_ were 0.7, 1.4, 2.1, and 10.5 nm, respectively.

### Raman single-spectrum characterization

A WITec Alpha 300 R was utilized to obtain the high-resolution spectra. A 532 nm laser was used to excite the PdSe_2_ flakes with a power of 0.25 mW, a 100× objective with NA = 0.9, and a laser spot size of 1 μm. The integration time was 5 s with accumulated ten times to obtain a high signal-to-noise ratio. The 1800 g mm^−1^ optical grating was used to improve the spectral resolution.

### SHG measurements and characterization

The reflective geometry with normal incidence excitation was used to achieve the SHG measurements. The ultrashort 80 fs pulses with a 80 MHz repetition rate (Spectra-Physics Solstice Maitai HP) were used as the excitation source for the SHG measurements. For SHG mapping characterization, the regions of 40 × 40 μm^2^ and 50 × 50 μm^2^ containing 1–8 L PdSe_2_ flakes were selected. A fiber-based pulsed laser was used as the fundamental pump source with the center wavelength at 800 nm. The laser pulses were focused onto a spot size of 6 μm on the PdSe_2_ sample by a 50× objective with NA = 0.55. The same objective lens was used to collect the SHG signal scattered from the sample and then detected by the charged coupling device (CCD) spectrometer. The integration time of 0.5 s and the scan duration of 2 h was set to achieve the high-resolution mapping images.

For the measurements of layer number dependent SHG signal, the excitation wavelength was fixed at 880 nm, and the same excitation intensity was used. For SHG polarization measurements, a half-wave plate (analyzer) was placed in the incident (outgoing) light path. Only the light with its polarization orientation parallel to the optical axis of the analyzer could pass and be detected by the CCD spectrometer. A step size of 10° was adopted for the parallel configuration.

### TPA and SA measurements

The TPA and SA of PdSe_2_ were measured using the nonlinear transmittance method in a home-built micro NLO system.The samples were excited at the wavelength of 600 nm (spectral width ~15 nm) or 800 nm (spectral width ~10 nm) by the laser pulses from an OPA combined with TOPAS (1000 Hz, 110 fs, Spectra-Physics, Inc.). The fs pulses were focused onto the sample by A 50× objective lens with NA = 0.55.

## Supplementary information

Supplementary Information

## Data Availability

All data generated during this study are included in this published article and its supplementary information files. All other relevant data are available from the corresponding authors on request.

## References

[1] Levenson M (1985). The principles of nonlinear optics. IEEE J. Quantum Electron..

[2] Barry RM, Robert WB (2009). Book review: nonlinear optics, 3rd edn. J. Biomed. Opt..

[3] Shen YR (1989). Surface properties probed by second-harmonic and sum-frequency generation. Nature.

[4] Bloembergen N (1982). Nonlinear optics and spectroscopy. Rev. Mod. Phys..

[5] Xia F, Mueller T, Lin Y-m, Valdes-Garcia A, Avouris P (2009). Ultrafast graphene photodetector. Nat. Nanotechnol..

[6] Ju L (2011). Graphene plasmonics for tunable terahertz metamaterials. Nat. Nanotechnol..

[7] Bao Q (2009). Atomic-layer graphene as a saturable absorber for ultrafast pulsed lasers. Adv. Funct. Mater..

[8] Tsai D-S (2013). Few-layer MoS2 with high broadband photogain and fast optical switching for use in harsh environments. ACS Nano.

[9] Liu W (2018). Tungsten diselenide for mode-locked erbium-doped fiber lasers with short pulse duration. Nanotechnology.

[10] Wang H, Qian X (2017). Giant optical second harmonic generation in two-dimensional multiferroics. Nano Lett..

[11] Zhou F, Ji W (2017). Giant three-photon absorption in monolayer MoS2 and its application in near-infrared photodetection. Laser Photon. Rev..

[12] Wang L (2019). Nonlinear optical signatures of the transition from semiconductor to semimetal in PtSe2. Laser Photon. Rev..

[13] Sun Z, Hasan T, Ferrari AC (2012). Ultrafast lasers mode-locked by nanotubes and graphene. Phys. E.

[14] Martinez A, Sun Z (2013). Nanotube and graphene saturable absorbers for fibre lasers. Nat. Photon..

[15] Wang J (2011). Graphene and carbon nanotube polymer composites for laser protection. J. Inorg. Organomet. Polym. Mater..

[16] Kumar N (2013). Third harmonic generation in graphene and few-layer graphite films. Phys. Rev. B.

[17] Nair RR (2008). Fine structure constant defines visual transparency of graphene. Science.

[18] Tran V, Soklaski R, Liang Y, Yang L (2014). Layer-controlled band gap and anisotropic excitons in few-layer black phosphorus. Phys. Rev. B.

[19] Li L (2017). Direct observation of the layer-dependent electronic structure in phosphorene. Nat. Nanotechnol..

[20] Lu SB (2015). Broadband nonlinear optical response in multi-layer black phosphorus: an emerging infrared and mid-infrared optical material. Opt. Express.

[21] Chen R, Tang Y, Zheng X, Jiang T (2016). Giant nonlinear absorption and excited carrier dynamics of black phosphorus few-layer nanosheets in broadband spectra. Appl. Opt..

[22] Mak KF, Shan J (2016). Photonics and optoelectronics of 2D semiconductor transition metal dichalcogenides. Nat. Photon..

[23] Oyedele AD (2017). PdSe2: Pentagonal two-dimensional layers with high air stability for electronics. J. Am. Chem. Soc..

[24] Choi Y (2020). Complete determination of the crystallographic orientation of ReX2 (X = S, Se) by polarized Raman spectroscopy. Nanoscale Horiz..

[25] Huanian Z (2020). Palladium selenide as a broadband saturable absorber for ultra-fast photonics. Nanophotonics.

[26] Nannan X (2020). Palladium diselenide as a direct absorption saturable absorber for ultrafast mode-locked operations: from all anomalous dispersion to all normal dispersion. Nanophotonics.

[27] Malard LM, Alencar TV, Barboza APM, Mak KF, de Paula AM (2013). Observation of intense second harmonic generation from MoS2 atomic crystals. Phys. Rev. B.

[28] Li Y (2013). Probing symmetry properties of few-layer MoS2 and h-BN by optical second-harmonic generation. Nano Lett..

[29] Zhang S (2015). Direct observation of degenerate two-photon absorption and its saturation in WS2 and MoS2 monolayer and few-layer films. ACS Nano.

[30] Li Y (2015). Giant two-photon absorption in monolayer MoS2. Laser Photon. Rev..

[31] Cirloganu CM, Padilha LA, Fishman DA, Webster S, Hagan DJ, Van Stryland ,EW (2011). Extremely nondegenerate two-photon absorption in direct-gap semiconductors [Invited]. Opt. Express.

[32] Zhang C (2019). Anisotropic nonlinear optical properties of a SnSe flake and a novel perspective for the application of all-optical switching. Adv. Optical Mater..

[33] Luo Z (2015). Nonlinear optical absorption of few-layer molybdenum diselenide (MoSe2) for passively mode-locked soliton fiber laser [Invited]. Photon. Res..

[34] Luo Z (2014). 1-, 1.5-, and 2-μm fiber lasers Q-switched by a broadband few-layer MoS2 saturable absorber. J. Lightwave Technol..

[35] Yuan H (2015). Polarization-sensitive broadband photodetector using a black phosphorus vertical p–n junction. Nat. Nanotechnol..

[36] Liang Q (2019). High-performance, room temperature, ultra-broadband photodetectors based on air-stable PdSe2. Adv. Mater..

[37] O’Brien M (2016). Mapping of low-frequency raman modes in CVD-grown transition metal dichalcogenides: layer number, stacking orientation and resonant effects. Sci. Rep..

[38] Ferrari AC (2006). Raman spectrum of graphene and graphene layers. Phys. Rev. Lett..

[39] Yu J (2020). Observation of double indirect interlayer exciton in WSe2/WS2 heterostructure. Opt. Express.

[40] Shi W (2016). Raman and photoluminescence spectra of two-dimensional nanocrystallites of monolayer WS2 and WSe2. 2D Mater..

[41] Soulard C (2004). Experimental and theoretical investigation on the relative stability of the PdS2- and pyrite-type structures of PdSe2. Inorg. Chem..

[42] Ribeiro-Soares J (2015). Second harmonic generation in WSe2. 2D Mater..

[43] Wang Y, Xiao J, Yang S, Wang Y, Zhang X (2019). Second harmonic generation spectroscopy on two-dimensional materials [Invited]. Opt. Mater. Express.

[44] Mennel L, Paur M, Mueller T (2018). Second harmonic generation in strained transition metal dichalcogenide monolayers: MoS2, MoSe2, WS2, and WSe2. APL Photon..

[45] Lin X (2018). Two-dimensional pyramid-like WS2 layered structures for highly efficient edge second-harmonic generation. ACS Nano.

[46] Song Y (2018). Extraordinary second harmonic generation in ReS2 atomic crystals. ACS Photon..

[47] Song Y (2018). Second harmonic generation in atomically thin MoTe2. Adv. Optical Mater..

[48] Wen X, Gong Z, Li D (2019). Nonlinear optics of two-dimensional transition metal dichalcogenides. InfoMat.

[49] Qiu X (2019). Strong multiphoton absorption in chiral CdSe/CdS dot/rod nanocrystal-doped poly(vinyl alcohol) films. Opt. Lett..

[50] Liu W (2017). Giant two-photon absorption and its saturation in 2D organic–inorganic perovskite. Adv. Optical Mater..

[51] Van Stryland EW, Woodall MA, Vanherzeele H, Soileau MJ (1985). Energy band-gap dependence of two-photon absorption. Opt. Lett..

[52] Drüppel M, Deilmann T, Krüger P, Rohlfing M (2017). Diversity of trion states and substrate effects in the optical properties of an MoS2 monolayer. Nat. Commun..

[53] Xie Y (2019). Layer-modulated two-photon absorption in MoS2: probing the shift of the excitonic dark state and band-edge. Photon. Res..

[54] Wang K (2013). Ultrafast saturable absorption of two-dimensional MoS2 nanosheets. ACS Nano.

[55] Wei C (2016). Passively Q-switched mid-infrared fluoride fiber laser around 3µm using a tungsten disulfide (WS2) saturable absorber. Laser Phys. Lett..

[56] Bikorimana S (2016). Nonlinear optical responses in two-dimensional transition metal dichalcogenide multilayer: WS2, WSe2, MoS2 and Mo0.5W0.5S2. Opt. Express.

[57] Chen B (2015). Q-switched fiber laser based on transition metal dichalcogenides MoS2, MoSe2, WS2, and WSe2. Opt. Express.

[58] Ren C (2019). A near-infrared I emissive dye: toward the application of saturable absorber and multiphoton fluorescence microscopy in the deep-tissue imaging window. Chem. Commun..

[59] Yuan J (2018). Few-layer platinum diselenide as a new saturable absorber for ultrafast fiber lasers. ACS Appl. Mater. Interfaces.

