# Supplementary material for: Giant nonlinear optical activity in two-dimensional palladium diselenide
Source: Nat Commun. 2021 Feb 17;12:1083. doi: 10.1038/s41467-021-21267-4 (PMC7889859; doi:10.1038/s41467-021-21267-4)
Supplement: Supplementary file 1 — Supplementary Information [file 41467_2021_21267_MOESM1_ESM.pdf]

Supplementary Information

Giant Nonlinear Optical Activity in Two-Dimensional Palladium Diselenide

Juan Yu[1,2,3,#], Xiaofei Kuang[3,#], Junzi Li[4,#],Jiahong Zhong[1,2], Cheng Zeng[1,2], Lingkai Cao[1,2], Zongwen Liu[5], Zhouxiaosong Zeng[6], Ziyu Luo[6], Tingchao He[4,*], Anlian Pan[6,*], & Yanping Liu[1,2,7*]

1. *School of Physics and Electronics, Hunan Key Laboratory for Super-microstructure and Ultrafast Process, Central South University, 932 South Lushan Road, Changsha, Hunan 410083, P. R. China*
2. *State Key Laboratory of High-Performance Complex Manufacturing, Central South University, 932 South Lushan Road, Changsha, Hunan 410083, P. R. China*
3. *School of Electronics and Information, Hangzhou Dianzi University,1158 Second Street, Xiasha College Park, Hangzhou, Zhejiang, 310018, P. R. China*
4. *College of Physics and Optoelectronic Engineering, Shenzhen University, Shenzhen 518060, P. R. China*
5. *School of Chemical and Biomolecular Engineering, The University of Sydney, NSW 2006, Australia*
6. *College of Materials Science and Engineering, Hunan University，Changsha, Hunan 410082, P. R. China*
7. *Shenzhen Research Institute of Central South University, A510a, High-tech Industrial Park, Yuehai Street, Shenzhen 518057, P. R. China*

* Correspondence and requests for materials should be addressed to Email: tche@szu.edu.cn; anlian.pan@hnu.edu.cn; liuyanping@csu.edu.cn

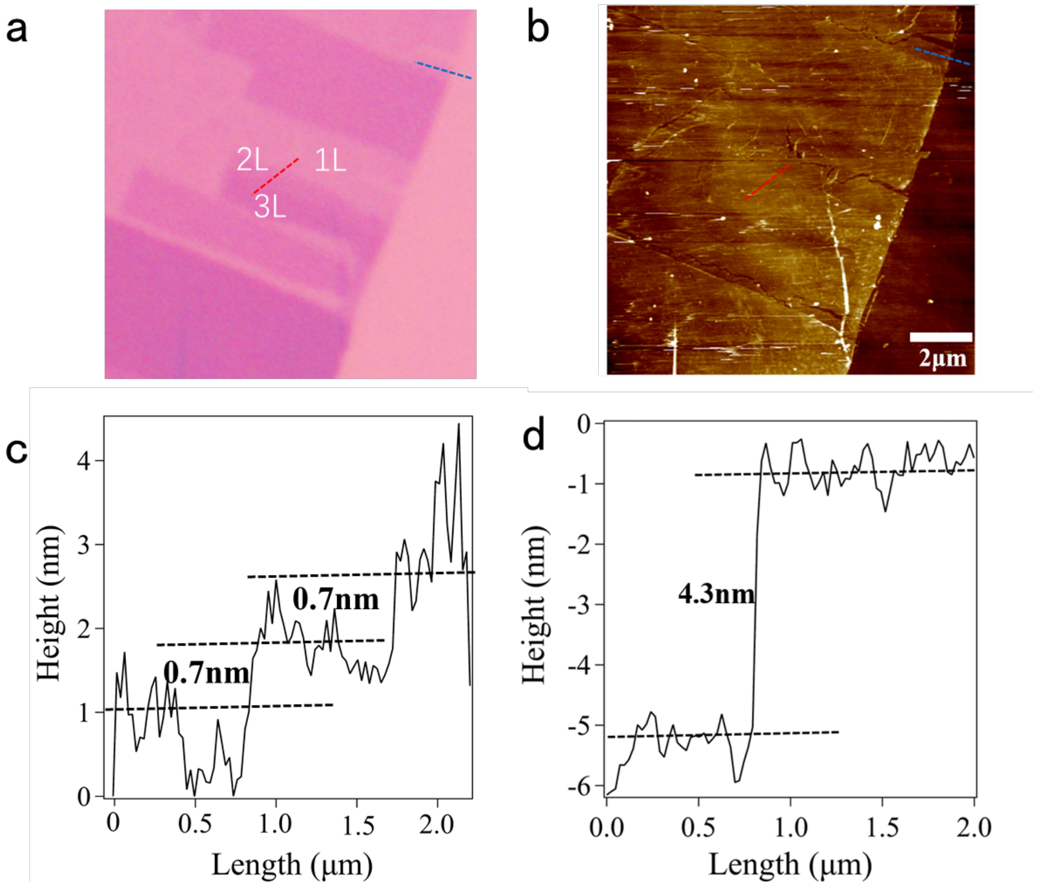

Figure S1. **Atomic force microscopic (AFM) investigation of few-layer $PdSe_2$ flakes.** (a) Optical microscope image of the few-layer $PdSe_2$ on the silicon substrate with a 285 nm SiO_2 layer. The number of sample layers containing 1-3 L was marked with white letters, and the scale bar is 2 μm. (b) The atomic force microscopic (AFM) images of the $PdSe_2$ corresponding to the region in (a). (c) The line profile scanned from the red line in the image (a) and (b) including 2 L and 3 L. The height difference between 1 L and 2 L is 0.7 nm. Meanwhile, the height difference between 2 L and 3 L is also 0.7 nm. (d) The line profile scanned from the blue line of monolayer $PdSe_2$ in the image (a) and (b). It shows 4.3 nm and not 0.7 nm, but the Raman spectrum indicates the monolayer, which confirms the difficulty to ascertain the very thin thickness of flakes from AFM.

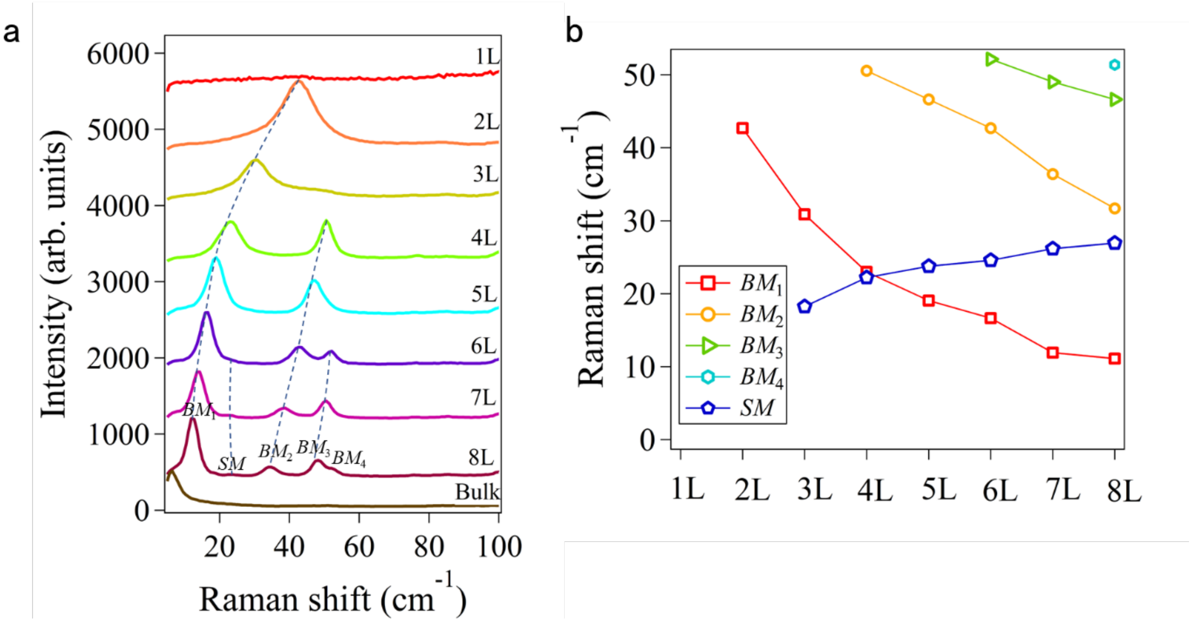

Figure S2: The low-frequency Raman shift of few-layer $PdSe_2$. (a) Raman spectra of 1-8 L and bulk $PdSe_2$. BM_1, BM_2, BM_3, and BM_4 are the first, second, third, and fourth branches of the breathing mode, respectively. *SM* is the shear mode of $PdSe_2$. The corresponding layer numbers are labeled on the right side of the panel. (b) Raman shift of BM_1, BM_2, BM_3, BM_4 and *SM* modes versus layer numbers. According to our experimental observations, the peak positions of the breathing and shear modes in different layers have a certain relationship with the layer numbers, as shown in (b). Therefore, the number of layers can be determined according to the peak positions of different modes. It can be seen that the peak position of BM_1, the first branch of breathing modes, changes more obviously with the number of layers (2L ~ 42.689 cm^{-1}, 3L ~ 30.8739 cm^{-1}, 4L ~ 22.9888 cm^{-1}, 5L ~ 19.0438 cm^{-1}, 6L ~ 16.6479 cm^{-1}, and 7L ~ 11.9246 cm^{-1}).

Note S1:

The second-order susceptibility $\chi^{(2)}$ of 4 L $PdSe_2$ is calculated by the following formula[1]:

$$I_{2\omega} = \left[\chi^{(2)}\right]^2 I_\omega^2 8\epsilon_0 c^3 \cdot \frac{1}{n_{2\omega} n_\omega^2} \cdot \frac{\omega^2 t^2}{8\epsilon_0 c^3} \quad \text{(S1)}$$

Where ϵ_0 is the vacuum dielectric constant, c is the vacuum light speed, $n_{2\omega}$ and n_ω are the refractive indexes of $PdSe_2$ at the SHG signal wavelength and the excitation wavelength[2,3]. ω is the frequency of the excitation light. t is the thickness of the sample. $I_{2\omega}$ and I_ω are the optical intensity of the SHG signal and incident light, which can be obtained by the following equations:

$$I_{2\omega} = \frac{2P_{2\omega}}{\epsilon_0 c\, \pi R_{2\omega}^2} \quad \text{(S2)}$$

$$I_\omega = \frac{2P_\omega}{\epsilon_0 c\, \pi R_\omega^2} \quad \text{(S3)}$$

Where $P_{2\omega}$ and P_ω are the power of the SHG signal and the incident light, respectively. And $R_{2\omega}$ and R_ω are the radius of the SHG signal and the excitation light. The second-order susceptibility $\chi^{(2)}$ of 4 L $PdSe_2$ is 5.17×10^{-11} m V^{-1}.

Table S1. Comparison of second-order nonlinear susceptibility in different 2D materials.

Material	$\chi^{(2)}$ m V^{-1}	Excitation wavelength	Thickness	Ref.
**ε-InSe**	1.3×10^{-11}	800 nm	~20 nm	1
**WS_2**	2.0×10^{-12}	800 nm	1L	
**ε-GaSe**	1.1×10^{-11}	800 nm	~20 nm	
**2H-$MoTe_2$**	2.5×10^{-9}	1550 nm	1L	4
**graphene**	9.0×10^{-11}	1300 nm	3L	5
**ReS_2**	9.0×10^{-10}	1558 nm	2L	6
**$PdSe_2$**	5.17×10^{-11}	880 nm	1L	This work

Table S1 represents the second-order nonlinear susceptibility ($\chi^{(2)}$) of several two-dimensional materials (including InSe, WS_2, GaSe, $MoTe_2$, $MoSe_2$, graphene, and ReS_2) and $PdSe_2$ (our work) under different laser excitation wavelength and sample thickness. The corresponding references are on the far right of the table.

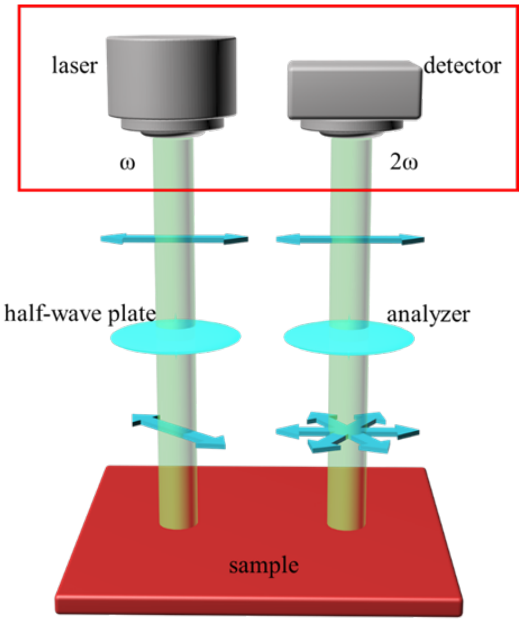

Figure S3. Schematic experimental configuration of the polarization-resolved SHG measurement in $PdSe_2$. The polarization direction of the incident light is regulated by the half-wave plate. And the polarization direction of the scattering light is determined by the analyzer. The microscopic imaging system to target the exact flakes is shown in the red box in the figure. The polarization-resolved SHG measurements were performed by our home-built micro-area nonlinear optical characterization system. The spot sizes of the fs pulses on the sample were ~6 μm at 880 nm, and the pump laser was supplied by Spectra-Physics Solstice Maitai HP (~80 fs pulses with 80 MHz repetition rate). During the measurement, the broadband ultrafast variable attenuators (Newport VA-BB) were employed to control the incident energy of the laser pulses. An analyzer was used to optimize with polarization to obtain the maximum the SHG signal. The SHG signals are collected by a 50× objective lens (NA = 0.55), and then detected by a spectrometer (SpectraPro HRS-300).

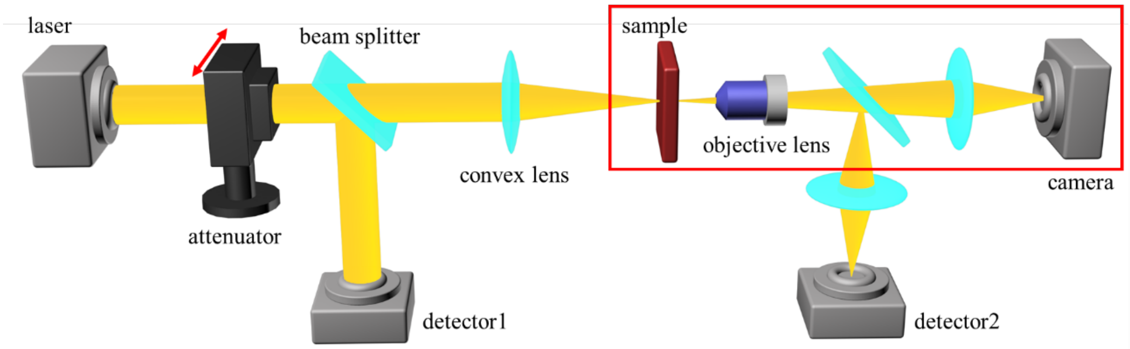

Figure S4. Schematic experimental setting of the I-scan measurement in $PdSe_2$, which is used for the TPA and SA measurement. The microscopic imaging system to target the exact flakes is shown in the red box in the figure. The TPA and SA coefficients of $PdSe_2$ were determined by our home-built micro-area nonlinear optical characterization system. The spot sizes of the fs pulses on the $PdSe_2$ were ~4 μm at 600 nm and ~6 μm at 800 nm, respectively. During the measurements, the broadband ultrafast variable attenuators (Newport VA-BB) were employed to control the incident energy of the laser pulses. The video microscope system (Olympus BX43) was constructed by the confocal microscope and the imaging development camera. The transmitted light passing through the $PdSe_2$ was first collected by a 50× objective lens with NA = 0.55 (working distance ~1 mm and focal length ~3.5 mm) and was then detected by a silicon detector.

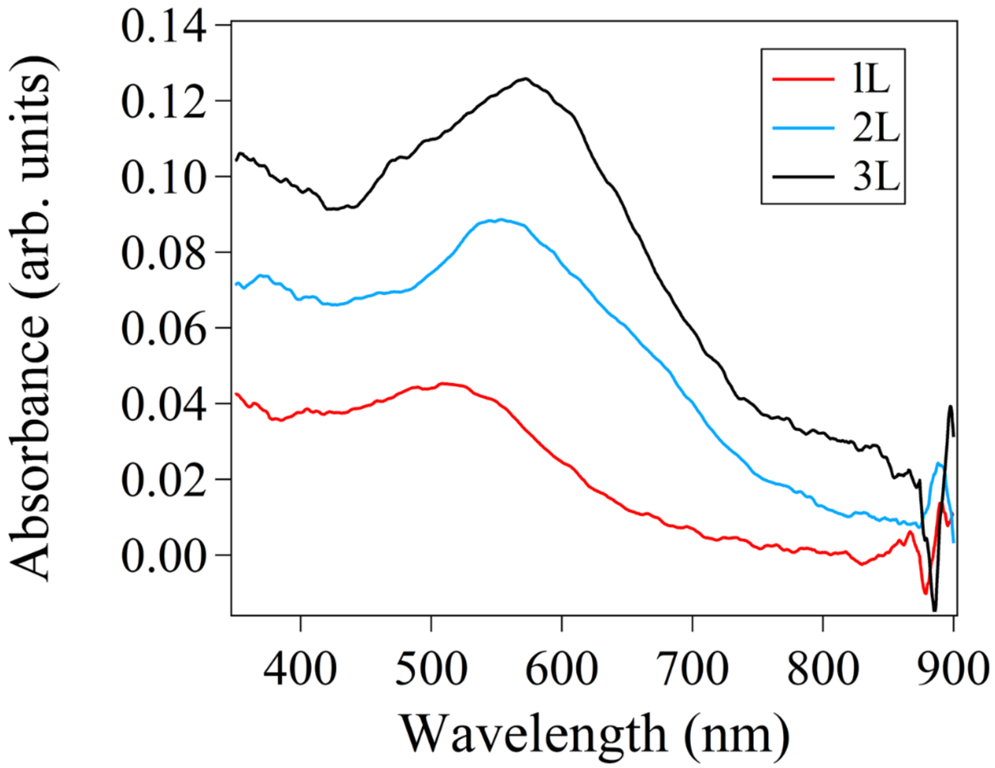

Figure S5. Linear absorption spectra of 1-3 L $PdSe_2$ flakes. A Jasco MSV 5200 microspectrophotometer was used to acquire high-resolution absorption spectra, which contains a microscopic system that can accurately define the sample area. The minimum measurement field size is 10 μm, and the magnification of the objective lens is ×32. The PMT detector was used for the visible region, and the NIR DET was utilized for the near-infrared region. The measured spectral range was 300−1000 nm. The data interval was 0.5 nm. The scanning speed was set to 1000 nm min^{-1}. Our samples of 1, 2, and 3 L $PdSe_2$ are beyond 40 μm. The linear absorption coefficient of 1, 2, and 3 L $PdSe_2$ at 800 nm are 3.9×10^3, 1.3×10^4, 1.9×10^4 cm^{-1}, respectively, according to the formula: $I = I_0 e^{-\mu t}$, where I_0 and I are the intensity of the incident and detecting light, μ is the linear absorption coefficient, and t is the thickness of the sample. The small linear absorption coefficient implies that TPA dominates the optical process under the large incident irradiance at 800 nm.

Table S2. Comparison of TPA coefficients in different 2D materials.

2D material	TPA coefficient (cm GW^{-1})	laser	thickness	Ref
MoS_2	4223 ± 62	1030nm,350fs,100Hz	1.5 ± 0.75 nm	7
	1065 ± 62		5.5 ± 0.75 nm	
	485 ± 11		18.7 ± 0.70 nm	
	499 ± 2		50.0 ± 0.75 nm	
$MoSe_2$	6906 ± 39	1030nm,350fs,100Hz	0.65 ± 0.65 nm	7
	4287 ± 58		1.4 ± 0.70 nm	
	2755 ± 133		5.8 ± 0.64 nm	
WS_2	4085 ± 67	1030nm,350fs,100Hz	1.20 ± 0.65 nm	7
	765 ± 44		7.5 ± 0.75 nm	
	788 ± 18		14.0 ± 0.70 nm	
	857 ± 20		30.0 ± 0.75 nm	
WSe_2	7102 ± 45	1030nm,350fs,100Hz	0.8 ± 0.65 nm	7
	3021 ± 47		1.6 ± 0.75 nm	
	2359 ± 201		5.6 ± 0.80 nm	
MoS_2	14200	1030nm,340fs,1kHz	1L	8
WS_2	6300		1L	
WS_2	10000 ± 8000	1030nm,340fs,1kHz	1-3L	9
	525 ± 205	800nm,40fs,1kHz	1-3L	
MoS_2	66 ± 4	1030nm,340fs,1kHz	25-27L	
	11.4 ± 4.3	800nm,40fs,1kHz	25-27L	
MoS_2	7500	532nm,10ns,1kHz	1L	10
MoS_2	70	800nm,100fs,1kHz	1L	11
MoS_2	7620 ± 150	1030nm,340fs,1kHz	1L	12
MoS_2	1880 ± 210	1030nm,340fs,1kHz	0.7 nm	13
$PdSe_2$	416000	800nm,110fs,1kHz	0.7 nm	This work
	258000		1.4 nm	
	151000		2.1 nm	
	18000		10.5 nm	

Table S2 represents the TPA coefficients of several conventional two-dimensional

materials (including MoS_2, $MoSe_2$, WS_2, and WSe_2) and $PdSe_2$ (our work) under different laser excitation and sample thickness. The corresponding references are on the far right of the table.

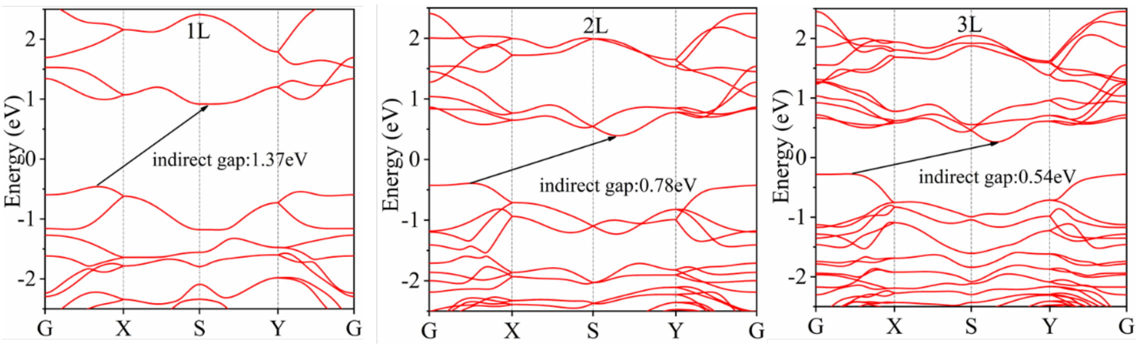

Figure S6. The theoretical bandgap of $PdSe_2$. Electronic band structure of 1 L (left panel), 2 L (middle panel), and 3 L (right panel) $PdSe_2$ calculated by DFT method. The minimum of the conduction band and the maximum of the valence band in each layer are indicated by the blue arrows. And the corresponding bandgaps of 1, 2 and 3 L $PdSe_2$ are 1.37, 0.78 and 0.54 eV, respectively. Theoretical calculation results exhibit that the indirect bandgap of $PdSe_2$ decreases with the increasing number of layers, from 1.37 eV of the monolayer to 0.03 eV of the bulk.

Reference:

1 Hao, Q., Yi, H., Su, H., Wei, B., Wang, Z., Lao, Z., Chai, Y., Wang, Z., Jin, C., Dai, J. & Zhang, W. Phase Identification and Strong Second Harmonic Generation in Pure ε-InSe and Its Alloys. *Nano Letters* **19**, 2634-2640 (2019).

2 Zhang, G., Amani, M., Chaturvedi, A., Tan, C., Bullock, J., Song, X., Kim, H., Lien, D.-H., Scott, M. C., Zhang, H. & Javey, A. Optical and electrical properties of two-dimensional palladium diselenide. *Applied Physics Letters* **114**, 253102 (2019).

3 Huanian, Z., Pengfei, M., Mingxiao, Z., Wenfei, Z., Guomei, W. & Shenggui, F. Palladium selenide as a broadband saturable absorber for ultrafast photonics. *Nanophotonics* **9**, 2557-2567 (2020).

4 Song, Y., Tian, R., Yang, J., Yin, R., Zhao, J. & Gan, X. Second Harmonic Generation in Atomically Thin MoTe2. *Advanced Optical Materials* **6**, 1701334 (2018).

5 Shan, Y., Li, Y., Huang, D., Tong, Q., Yao, W., Liu, W.-T. & Wu, S. Stacking symmetry governed second harmonic generation in graphene trilayers. *Science Advances* **4**, eaat0074 (2018).

6 Song, Y., Hu, S., Lin, M.-L., Gan, X., Tan, P.-H. & Zhao, J. Extraordinary Second Harmonic Generation in ReS2 Atomic Crystals. *ACS Photonics* **5**, 3485-3491 (2018).

7 Dong, N., Li, Y., Zhang, S., McEvoy, N., Gatensby, R., Duesberg, G. S. & Wang, J. Saturation of Two-Photon Absorption in Layered Transition Metal Dichalcogenides: Experiment and Theory. *ACS Photonics* **5**, 1558-1565 (2018).

8 Dai, X., Zhang, X., Kislyakov, I. M., Wang, L., Huang, J., Zhang, S., Dong, N. & Wang, J. Enhanced two-photon absorption and two-photon luminescence in monolayer MoS2 and WS2 by defect repairing. *Opt. Express* **27**, 13744-13753 (2019).

9 Zhang, S., Dong, N., McEvoy, N., O'Brien, M., Winters, S., Berner, N. C., Yim, C., Li, Y., Zhang, X., Chen, Z., Zhang, L., Duesberg, G. S. & Wang, J. Direct Observation of Degenerate Two-Photon Absorption and Its Saturation in WS2 and MoS2 Monolayer and Few-Layer Films. *ACS Nano* **9**, 7142-7150 (2015).

10 Zhang, J., Ouyang, H., Zheng, X., You, J., Chen, R., Zhou, T., Sui, Y., Liu, Y., Cheng, X. a. & Jiang, T. Ultrafast saturable absorption of MoS2 nanosheets under different pulse-width excitation conditions. *Opt. Lett.* **43**, 243-246 (2018).

11 Zhou, F. & Ji, W. Two-photon absorption and subband photodetection in monolayer MoS2. *Opt. Lett.* **42**, 3113-3116 (2017).

12 Li, Y., Dong, N., Zhang, S., Zhang, X., Feng, Y., Wang, K., Zhang, L. & Wang, J. Giant two-photon absorption in monolayer MoS2. *Laser & Photonics Reviews* **9**, 427-434 (2015).

13 Xie, Y., Zhang, S., Li, Y., Dong, N., Zhang, X., Wang, L., Liu, W., Kislyakov, I. M., Nunzi, J.-M., Qi, H., Zhang, L. & Wang, J. Layer-modulated two-photon absorption in MoS2: probing the shift of the excitonic dark state and band-edge. *Photon. Res.* **7**, 762-770 (2019).
